# The prognostic value of preoperative systemic inflammatory response index in predicting outcomes of acute type A aortic dissection patients underwent surgical treatment

**DOI:** 10.3389/fimmu.2024.1388109

**Published:** 2024-05-10

**Authors:** Lin-feng Xie, Qi-gui Xie, Wen-ping Gao, Qing-song Wu, Xin-fan Lin, Zhi-huang Qiu, Liang-wan Chen

**Affiliations:** ^1^ Department of Cardiovascular Surgery, Fujian Medical University Union Hospital, Fuzhou, Fujian, China; ^2^ Key Laboratory of Cardio-Thoracic Surgery (Fujian Medical University), Fujian Province University, Fuzhou, Fujian, China; ^3^ Fujian Provincial Center for Cardiovascular Medicine, Fuzhou, Fujian, China; ^4^ The Affiliated Longyan First Hospital of Fujian Medical University, Longyan, Fujian, China

**Keywords:** systemic inflammatory response index, acute type A aortic dissection, major adverse events, aorta-related adverse events, prognostic marker

## Abstract

**Background:**

The systemic inflammatory response index (SIRI) is a novel inflammatory-immune biological marker that has prognostic value in various cardiovascular diseases. This study aims to investigate the relationship between SIRI and short-term and long-term prognosis in patients with acute type A aortic dissection (AAAD) underwent surgical treatment.

**Methods:**

We conducted a retrospective analysis of patients with AAAD who underwent emergency surgical treatment at our center. Through multifactorial logistics regression analysis and cox proportional hazards regression analysis, we identified SIRI as an independent risk factor for major adverse events (MAEs) and long-term aorta-related adverse events (ARAEs) post-surgery. The optimal cutoff value of preoperative SIRI was determined using receiver operating characteristic (ROC) curve analysis, and patients were divided into low SIRI group and high SIRI group. The prognostic outcomes at different time points post-surgery for the two groups of patients were analyzed using Kaplan-Meier survival analysis, and the significance was determined by log-rank test.

**Results:**

A total of 691 AAAD patients were included in this study. Among them, 50 patients (7.2%) died within 30 days post-surgery, and 175 patients (25.3%) experienced MAEs. A total of 641 patients were followed up, with an average follow-up time of 33.5 ± 17.5 months, during which 113 patients (17.6%) experienced ARAEs. The results of multifactorial logistics regression analysis and cox proportional hazards regression analysis showed that SIRI was an independent risk factor for postoperative MAEs (OR=3.148, 95%CI[1.650-6.006], *p*<0.001) and ARAEs (HR=2.248, 95%CI[1.050-4.809], *p*<0.037). Kaplan-Meier analysis demonstrated that the MAEs-free survival in the high SIRI group was significantly lower than that in the low SIRI group, and a similar trend was observed in the ARAEs-free survival during follow-up (log-rank test, *p*<0.001).

**Conclusion:**

Preoperative SIRI is significantly associated with the short-term and long-term prognosis of AAAD patients underwent emergency open surgery, demonstrating its valuable prognostic value. Therefore, preoperative SIRI is a reliable biological marker that can serve as a valuable tool for preoperative risk stratification and decision management.

## Introduction

Acute type A aortic dissection (AAAD) is a life-threatening cardiovascular emergency characterized by sudden onset and rapid progression. If not treated promptly, the mortality rate increases by approximately 1%-2% per hour, with a mortality rate of up to 50% within 24 hours (1–3). Surgery to repair the diseased aorta is the only effective way to save the lives of patients with AAAD. However, due to the severity of the condition before surgery and the difficulty of the operation, the rate of postoperative complications and mortality remains high, and the prognosis is poor (4). Research has reported that the mortality rate of AAAD patients after emergency open surgery is about 10%-25%, which is much higher than other types of cardiovascular surgery (5). Therefore, if some biomarkers can be used to assist clinicians in risk stratification of AAAD patients before surgery, high-risk patients with poor prognosis can be identified early, and appropriate treatment strategies can be developed to improve postoperative survival.

Inflammation has been confirmed to play a significant role in various cardiovascular diseases, and as the most important pathophysiological change throughout the development of aortic dissection (AD), the inflammatory response is closely related to the occurrence and progression of AD (6). Studies have reported significant changes in the levels of inflammatory and immune-related biomarkers in the peripheral blood of AD patients (such as white blood cells, neutrophils, monocytes, lymphocytes, and C-reactive protein). Moreover, increasing clinical evidence indicates that these inflammatory and immune-related biomarkers are closely associated with the prognosis of AD (7). However, due to the limited predictive value of single indicators, novel composite biomarkers combining multiple indicators [such as neutrophil-to-lymphocyte ratio (NLR), monocyte-to-lymphocyte ratio (MLR), and platelet-to-lymphocyte ratio (PLR)] can further enhance their prognostic value. These composite biomarkers are easy to obtain results for, less influenced by physiological conditions, provide more comprehensive information, and offer more valuable references for the prognosis of AD.

Recently, the systemic inflammatory response index (SIRI) has been discovered as a more comprehensive inflammatory and immune biomarker that integrates changes in multiple cell types, better representing the current level of inflammation and immune status of the body. Its potential predictive value has been confirmed in many diseases, especially in cardiovascular diseases (8, 9). However, few studies have investigated the impact of SIRI on AAAD patients receiving surgical treatment. Therefore, this study aims to clarify the effect of SIRI on postoperative prognosis of AAAD patients through data analysis from a large sample size in a single center.

## Materials and methods

### Study population

This study included a total of 691 AAAD patients who underwent emergency surgical treatment at our center from January 2018 to January 2022. The inclusion criteria were: (1) age>18 years; (2) confirmed diagnosis of Stanford type A aortic dissection by thoracic aortic angiography or echocardiography; (3) underwent emergency open surgical treatment after admission. The exclusion criteria were: (1) patients who had previously undergone open chest surgery; (2) patients with conditions that could affect inflammatory cell counts, including but not limited to active malignant tumors, acute infections, long-term use of drugs affecting blood cell counts, hematological disorders, and autoimmune diseases. All patients had venous blood samples taken for blood tests in a medication-free state before emergency surgery.

### Outcomes and definitions

This study’s primary outcome events included major adverse events (MAEs) during hospitalization and aortic-related adverse events (ARAEs) during follow-up. Evaluated MAEs included major complications during the perioperative period and all-cause mortality within 30 days postoperatively. According to the grading criteria for complications in postoperative clinical outcomes consensus for aortic arch surgery, the major postoperative complications were determined to include (10): (1) cardiovascular complications [myocardial infarction, malignant arrhythmias, and heart failure requiring mechanical support with an intra-aortic balloon pump (IABP)]; (2) pulmonary complications (acute lung injury, acute respiratory distress syndrome, prolonged mechanical ventilation, tracheostomy and respiratory failure requiring extracorporeal membrane oxygenation (ECMO) treatment); (3) new-onset acute kidney injury (serum creatinine levels increased more than 3 times the baseline value, absolute increase requiring prolonged mechanical ventilation or CRRT for renal failure); (4) wound complications requiring reoperation or requiring re-thoracotomy for hemostasis; (5) gastrointestinal bleeding. ARAEs included leakage during follow-up, distal aortic expansion > 5mm, progression of aortic disease, and aortic-related mortality.

### Data collection

The preoperative variables in this study include: gender, age, body mass index (BMI), left ventricular ejection fraction (LVEF), smoking and alcohol history, hypertension, diabetes, previous coronary artery disease (CAD), previous cerebrovascular disease (CVD), previous chronic kidney disease (CKD), Marfan syndrome, aortic valve regurgitation (moderate or above), pericardial effusion (moderate or above), white blood cells, platelets, hemoglobin, alanine transaminase (ALT), aspartate transaminase (AST), albumin (ALB), creatinine, D-dimer, fibrinogen, B-type natriuretic peptide (BNP), neutrophil-to-lymphocyte ratio (NLR), monocyte-to-lymphocyte ratio (MLR), platelet-to-lymphocyte ratio (PLR), systemic immune inflammation index (SII), systemic inflammatory response index (SIRI).

NLR = neutrophil count/lymphocyte count, MLR = monocyte count/lymphocyte count, PLR = platelet count/lymphocyte count, SII = platelet count * NLR. SIRI = monocyte count * NLR. Perioperative data recorded in this study include the main surgical procedure, total operation time, cardiopulmonary bypass (CPB) time, aortic cross clamp (ACC) time, deep hypothermic circulatory arrest (DHCA) time, and intraoperative blood product transfusion situation (red blood cell (RBC), plasma, platelet). All data are sourced from the hospital’s electronic medical record system. Follow-up data are assessed based on the final medical records or telephone interviews.

### Statistical analysis

The data were statistically processed and analyzed using SPSS 25.0 and R 4.2.1. Continuous variables were compared using Student’s t-test or Mann-Whitney U test, and the results were presented as mean ± standard deviation (SD) or median [interquartile range (IQR)]. Categorical variables were compared using chi-square test or Fisher’s test, and the results were presented as frequencies or percentages. Univariate analysis was performed to analyze the factors associated with MAEs and ARAEs. Variables with statistically significant differences in the univariate analysis were further included in multivariable logistics regression analysis and cox proportional hazards regression analysis to determine independent risk factors for postoperative MAEs and ARAEs. The accuracy of SIRI in predicting MAEs and ARAEs was evaluated using receiver operating characteristic (ROC) analysis. The results were presented as area under the curve (AUC), and the optimized cutoff value, sensitivity, and specificity were recorded. Additionally, the cutoff point corresponding to the maximum value of the Youden index was chosen as the optimal cutoff value for preoperative SIRI. Based on the optimized cutoff value of SIRI, patients were divided into high SIRI group and low SIRI group. The clinical outcomes after surgery were compared between the two groups. Kaplan-Meier survival curves were used to compare short-term and long-term prognostic outcomes after surgery, and their significance was determined using the log-rank test. A two-sided *p* value < 0.05 was considered statistically significant.

## Results

### Patient characteristics

**Figure 1:**
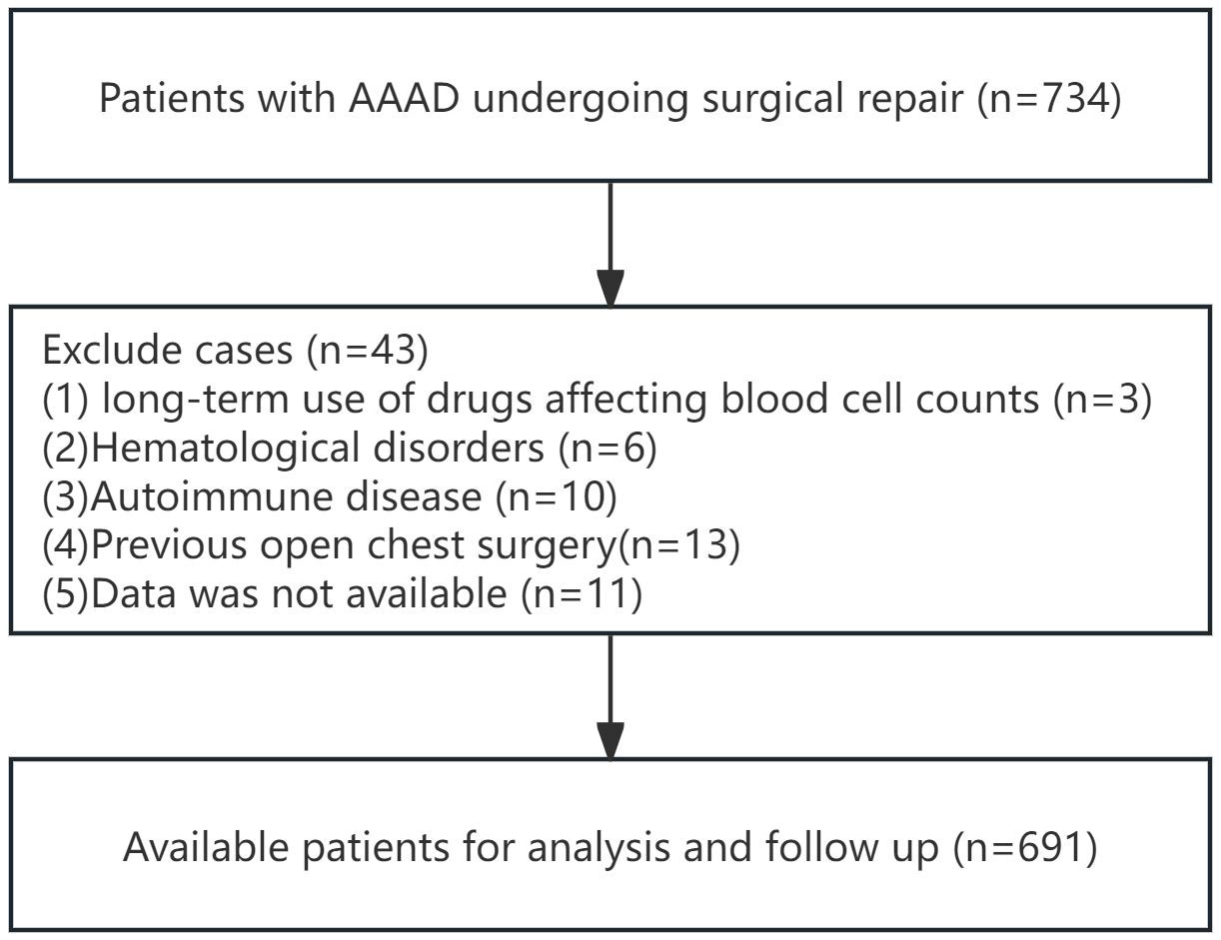
Flow chart for patients inclusion and exclusion of this study.

**Figure 2:**
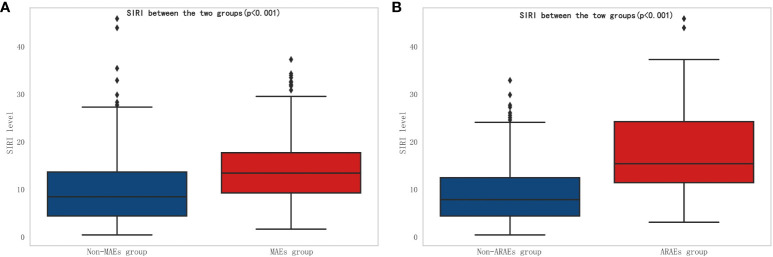
Comparison of SIRI between different groups. **(A)** Comparison of SIRI between non-MAEs group and MAEs group; **(B)** Comparison of SIRI between non-ARAEs group and ARAEs group. (SIRI, Systemic inflammatory response index; MAEs, Major adverse events; ARAEs, aorta-related adverse events).

According to the inclusion and exclusion criteria for patient selection, the workflow chart is shown in **Figure 1**. A total of 691 patients were included in this study, with 438 males (63.4%) and the average age of 53.2 ± 11.8 years. In this study, there were 175 cases (25.3%) of MAEs, and 50 cases (7.2%) of all-cause mortality within 30 days. The perioperative characteristics of patients in the MAEs and non-MAEs groups are shown in **Supplementary Table 1**. A total of 641 patients participated in follow-up, with the average follow-up time of 33.5 ± 17.5 months, and 113 cases (17.6%) experienced ARAEs during follow-up. The perioperative characteristics of patients in the ARAEs and non-ARAEs groups are shown in **Supplementary Table 2**. The SIRI level increased significantly in both the MAEs and ARAEs groups compared to their respective non-event groups (**Figure 2**).

### Logistic regression analysis and cox proportional hazards regression analysis

To further clarify the clinical variables associated with MAEs, we used logistic regression analysis to identify independent risk factors for postoperative MAEs in patients with AAAD undergoing surgical treatment. The results showed that PLT (*p*=0.006), ALB (*p*=0.007), creatinine (*p*=0.001), SII (*p*=0.029), SIRI (*p*=0.001), and operative time (*p*=0.001) were independent risk factors for postoperative MAEs in AAAD patients (**Table 1**). Subsequently, we used Cox proportional hazards regression analysis to determine independent predictors of ARAEs during follow-up. The results of the multivariable Cox regression analysis showed that Fibrinogen (*p*=0.009) and SIRI (*p*=0.037) were independent risk factors for ARAEs (**Table 2**).

**Table 1:** Univariate and multivariate logistics regression analysis for perioperative characteristics predictive of postoperative MAEs.

Valuables	Univariate analysis				Multivariate analysis			
	OR	95% CI		P value	OR	95% CI		P value
		Lower	Upper			Lower	Upper	
WBC (>13.86×10ˆ9/L)	2.190	1.541	3.113	<0.001	–	–	–	–
PLT (≤173×10ˆ9/L)	2.006	1.418	2.840	<0.001	1.937	1.210	3.098	0.006
ALT (>45IU/L)	1.947	1.308	2.897	0.001	–	–	–	–
ALB (≤39g/L)	1.910	1.325	2.754	0.001	1.825	1.182	2.817	0.007
Creatinine (>131μmol/L)	3.457	2.271	5.261	<0.001	2.347	1.412	3.900	0.001
D-dimer (>7.319μg/mL)	2.049	1.417	2.964	<0.001	–	–	–	–
Fibrinogen (≤2.21g/L)	1.961	1.379	2.789	<0.001	–	–	–	–
BNP (>165pg/mL)	1.601	1.101	2.329	0.014	–	–	–	–
NLR (>13.413)	2.499	1.748	3.572	<0.001	–	–	–	–
MLR (>0.773)	3.146	2.151	4.603	<0.001	–	–	–	–
SII (>2783.016)	1.830	1.292	2.593	0.001	1.839	1.064	3.178	0.029
SIRI (>10.764)	3.031	2.125	4.323	<0.001	3.148	1.650	6.006	0.001
Operative time (>309min)	3.836	2.359	6.237	<0.001	2.891	1.524	5.486	0.001
CPB time (>172min)	1.882	1.292	2.741	0.001	–	–	–	–

OR, Hazard ratio; CI, Confidence interval; MAEs, Major adverse events; WBC, White blood cell; PLT, Platelet; ALT, Alanine transaminase; ALB, Albumin; BNP, B-type natriuretic peptide; NLR, Neutrophil-to-lymphocyte ratio; MLR, Monocyte-to-lymphocyte ratio; SII, Systemic immune inflammation index; SIRI, Systemic inflammatory response index; CPB, Cardiopulmonary bypass.

**Table 2:** Univariate and multivariable cox proportional hazards regression analyses for perioperative characteristics predictive of ARAEs.

Valuables	Univariate analysis				Multivariate analysis			
	HR	95% CI		P value	HR	95% CI		P value
		Lower	Upper			Lower	Upper	
WBC (>15.33×10ˆ9/L)	2.525	1.728	3.688	<0.001	–	–	–	–
D-dimer (>6.93μg/mL)	1.624	1.085	2.430	0.018	–	–	–	–
Fibrinogen (≤2.16g/L)	1.784	1.230	2.587	0.002	1.739	1.145	2.64	0.009
NLR (>14.198)	3.764	2.467	5.744	<0.001	–	–	–	–
MLR (>0.892)	4.684	2.976	7.373	<0.001	–	–	–	–
PLR (>237.255)	2.285	1.56	3.347	<0.001	–	–	–	–
SII (>3087.391)	3.158	2.177	4.580	<0.001	–	–	–	–
SIRI (>10.764)	5.673	3.555	9.053	<0.001	2.248	1.050	4.809	0.037

HR, Hazard ratio; CI, Confidence interval; ARAE, aorta-related adverse events; WBC, White blood cells; NLR, Neutrophil-to-lymphocyte ratio; MLR, Monocyte-to-lymphocyte ratio; PLR, Platelets-to-lymphocyte ratio; SII, Systemic immune inflammation index; SIRI, systemic inflammatory response index.

### Sensitivity and specificity of SIRI in predicting prognosis outcomes

**Figure 3:**
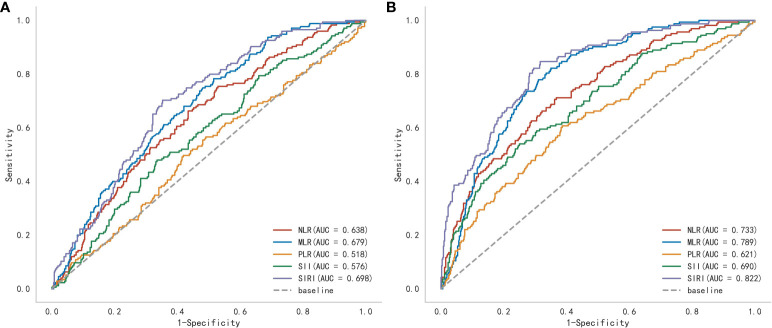
A receiver operating characteristic (ROC) curve to compare the value of several preoperative inflammatory biomarkers to identify postoperative **(A)** major adverse events and **(B)** aorta-related adverse events. (NLR, Neutrophil-to-lymphocyte ratio; MLR, Monocyte-to-lymphocyte ratio; PLR, Platelet-to-lymphocyte ratio; SII, Systemic immune inflammation index; SIRI, Systemic inflammatory response index).

Through ROC curve analysis, the optimal cutoff value of SIRI in predicting postoperative MAEs and ARAEs was determined (**Figure 3**). The results showed that compared to other composite inflammatory immune indicators, SIRI performed the best in predicting postoperative MAEs in AAAD patients, with an AUC of 0.698, an optimal cutoff value of 10.764, a sensitivity of 70.3%, and a specificity of 64.7% (**Supplementary Table 3**). We also verified the predictive value of SIRI for the long-term prognosis of AAAD patients after surgery. The results indicated that SIRI continued to demonstrate the best predictive performance, with an AUC of 0.822, an optimal cutoff value of 10.764, a sensitivity of 84.7%, and a specificity of 68.4% (**Supplementary Table 4**).

### Comparison of clinical outcomes between the high SIRI and low SIRI groups

**Figure 4:**
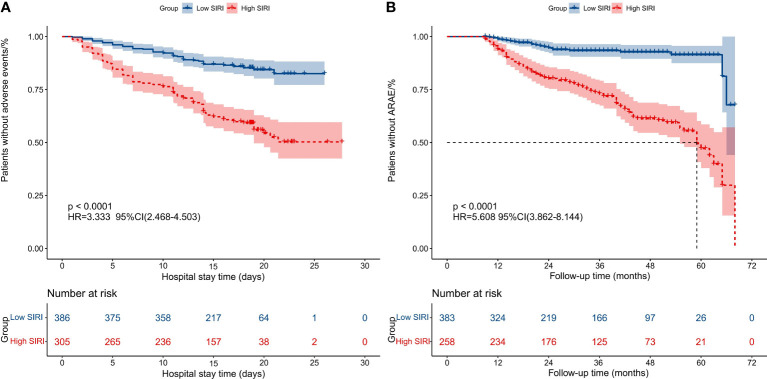
Kaplan-Meier analysis compared the survival condition for MAEs-free and ARAEs-free among different SIRI groups. **(A)** Kaplan-Meier analysis compared the survival condition for MAEs-free among two groups; **(B)** Kaplan-Meier analysis compared the survival condition for ARAEs-free among two groups. (SIRI, Systemic inflammatory response index; MAEs, Major adverse events; ARAEs, aorta-related adverse events).

To further evaluate the impact of SIRI on prognosis outcomes in AAAD patients, we chose the optimal cutoff value for SIRI as 10.764 and divided patients into low SIRI group (<10.764) and high SIRI group (>10.764). The comparison of preoperative and intraoperative conditions for both groups of patients is shown in **Table 3**. We compared the prognosis outcomes of the two groups of patients and found that the incidence of postoperative MAEs was significantly higher in the high SIRI group than in the low SIRI group. The incidence of postoperative complications such as CRRT treatment, ECOM treatment, acute kidney injury, permanent neurological dysfunction, low cardiac output syndrome, and gastrointestinal bleeding also increased significantly in the high SIRI group. In addition, the hospital stay time, ICU stay time, and mechanical ventilation time were significantly prolonged in the high SIRI group (**Table 4**). The Kaplan-Meier analysis in **Figure 4** compared the survival condition for MAEs-free and ARAEs-free among different SIRI groups. The results showed that the MAEs-free survival in the high SIRI group was significantly lower than that in the low SIRI group, and a similar trend was observed in the ARAEs-free survival during follow-up (log-rank test, *p*<0.001).

**Table 3:** Subgroup analysis of the correlation between SIRI and postoperative MAEs.

Valuables	N	SIRI		P value	P value for interaction
		Low	High		
PLT					0.668
≤173×10ˆ9/L	306	1.0 (ref.)	4.939 [2.947,8.277]	<0.001	
>173×10ˆ9/L	385	1.0 (ref.)	4.184 [2.405,7.279]	<0.001	
ALB					0.099
≤39g/L	404	1.0 (ref.)	3.540 [2.263,5.539]	<0.001	
>39g/L	287	1.0 (ref.)	7.212 [3.523,14.763]	<0.001	
Creatinine					0.400
≤131μmol/L	578	1.0 (ref.)	4.650 [3.002,7.203]	<0.001	
>131μmol/L	113	1.0 (ref.)	3.174 [1.463,6.885]	0.003	
SII					0.338
≤2783.016	434	1.0 (ref.)	4.818 [2.958,7.847]	<0.001	
>2783.016	257	1.0 (ref.)	3.238 [1.691,6.202]	<0.001	
Operative time					0.610
≤309min	614	1.0 (ref.)	4.167 [2.761,6.291]	<0.001	
>309min	77	1.0 (ref.)	5.492 [2.067,14.597]	0.001	

MAEs, Major adverse events; PLT, Platelet; ALB, Albumin; SII, Systemic immune inflammation index; SIRI, Systemic inflammatory response index.

**Table 4:** Subgroup analysis of the correlation between SIRI and ARAEs during follow-up.

Valuables	N	SIRI		P value	P value for interaction
		Low	High		
Fibrinogen					0.986
≤2.16g/L	212	1.0 (ref.)	6.214 [3.110,12.418]	<0.001	
>2.16g/L	429	1.0 (ref.)	5.759 [2.996,11.070]	<0.001	

ARAEs, aorta-related adverse events; SIRI, Systemic inflammatory response index.

### Subgroup analysis

Subgroup analysis was conducted to determine the association between SIRI and different clinical indicators, as well as its impact on prognosis outcomes. The results are shown in **Tables 5**, **6**. There was no significant difference in the relationship between SIRI and MAEs or ARAEs among different subgroups (interaction *p>*0.05). Across all subgroups, an increase in SIRI levels was closely associated with an increase in postoperative MAEs and ARAEs in AAAD patients

**Table 5:** The comparison of preoperative and intraoperative conditions in different SIRI groups.

Valuables	Low SIRI group (n=386)	High SIRI group (n=305)	P value
Demographical data			
Age (years), median[IQR]	53.00[45.00,62.00]	54.00[45.00,63.00]	0.519
Gender (Male), n (%)	252 (65.28)	186 (60.98)	0.244
Body mass index (Kg/mˆ2), median[IQR]	24.49[22.49,26.67]	24.35[22.96,26.17]	0.949
LVEF (%), median[IQR]	64.20[60.50,67.90]	63.60[60.10,66.20]	0.049
Risk factors and comorbidities			
Smoking, n (%)	175 (45.34)	134 (43.93)	0.713
Alcohol, n (%)	60 (15.54)	58 (19.02)	0.228
Hypertension, n (%)	286 (74.09)	225 (73.77)	0.924
Diabetes, n (%)	9 (2.33)	11 (3.61)	0.321
Previous CAD, n (%)	1 (0.26)	3 (0.98)	0.213
Previous CVD, n (%)	9 (2.33)	11 (3.61)	0.321
Previous CKD, n (%)	6 (1.55)	2 (0.66)	0.273
Marfan Syndrome, n (%)	7 (1.81)	8 (2.62)	0.468
Pericardial effusion (Medium or above), n (%)	20 (5.18)	32 (10.49)	0.009
Aortic valve regurgitation (Medium or above), n (%)	92 (23.83)	59 (19.34)	0.156
Preoperative laboratory results			
WBC (×10ˆ9/L), median[IQR]	10.84[8.65,13.04]	14.18[11.64,17.13]	<0.001
HB (g/L), median[IQR]	131.00[119.00,142.00]	131.00[119.00,144.00]	0.677
PLT (×10ˆ9/L), median[IQR]	179.00[148.00,219.00]	179.00[145.00,215.00]	0.734
ALT (U/L), median[IQR]	25.00[15.00,39.00]	26.00[17.00,39.00]	0.359
AST (U/L), median[IQR]	25.00[20.00,41.00]	26.00[18.00,51.00]	0.756
ALB (g/L), median[IQR]	38.10[35.00,40.90]	38.20[34.70,40.80]	0.711
Creatinine (μmol/L), median[IQR]	76.00[63.40,104.00]	80.00[56.00,118.00]	0.915
D-dimer (ug/mL), median[IQR]	8.91[3.39,18.57]	9.23[4.64,20.00]	0.183
Fibrinogen, median[IQR]	2.66[1.93,3.95]	2.69[1.95,3.54]	0.636
BNP (pg/mL), median[IQR]	256.00[117.00,630.00]	257.00[134.00,601.00]	0.510
NLR, median[IQR]	10.13[6.26,14.45]	17.53[12.97,24.85]	<0.001
MLR, median[IQR]	0.60[0.44,0.76]	1.38[1.05,1.75]	<0.001
PLR, median[IQR]	190.54[136.78,258.90]	266.06[185.06,382.98]	<0.001
SII, median[IQR]	1725.01[1097.90,2588.18]	3087.39[2160.09,4693.84]	<0.001
Intraoperative conditions			
Ascending aorta replacement, n (%)	385 (99.74)	305 (100.00)	1.000
**Root surgery**			0.529
Untreated	144 (37.31)	113 (37.05)	
Reconstruction of sinus of valsava	160 (41.45)	123 (40.33)	
Bentall	75 (19.43)	56 (18.36)	
Wheat	5 (1.30)	11 (3.61)	
David	1 (0.26)	1 (0.33)	
CABG (n, %)	13 (3.37)	11 (3.61)	0.865
Mitral surgery (n, %)	6 (1.55)	4 (1.31)	0.791
TVP (n, %)	7 (1.81)	4 (1.31)	0.601
Operation time (min), median[IQR]	254.00[225.44,281.00]	245.00[228.00,285.00]	0.888
CPB time (min), median[IQR]	153.00[137.56,175.00]	148.00[137.07,171.00]	0.427
ACC time (min), median[IQR]	60.00[45.00,78.00]	60.00[52.96,77.00]	0.040
DHCA time (min), median[IQR]	13.00[12.00,13.00]	13.00[12.00,14.00]	0.923
Plasma transfusion volume (mL), median[IQR]	200.00[200.00,350.00]	250.00[200.00,400.00]	0.083
RBC transfusion volume (U), median[IQR]	3.50[0.00,4.00]	4.00[2.00,4.00]	0.253
Platelet transfusion volume (U), median[IQR]	1.00[0.80,10.00]	2.00[1.00,10.00]	0.068

IQR, Interquartile range; LVEF, Left ventricular ejection fraction; CAD, Coronary artery disease; CVD, Cerebrovascular disease; CKD, Chronic kidney disease; WBC, White blood cell; HB, Heamoglobin; PLT, Platelet; ALT, Alanine transaminase; AST, Aspartate transaminase; ALB, Albumin; BNP, B-type natriuretic peptide; NLR, Neutrophil-to-lymphocyte ratio; MLR, Monocyte-to-lymphocyte ratio; PLR, Platelets-to-lymphocyte ratio; SII, Systemic immune inflammation index; SIRI, Systemic inflammatory response index; CABG, Coronary artery bypass grafting; TVP, Tricuspid valvuloplasty; CPB, Cardiopulmonary bypass; ACC, Aortic cross clamp; DHCA, Deep hypothermic circulatory arrest; RBC, Red blood cell.

**Table 6:** Short- and long-Term Outcomes of different SIRI group.

Valuables	Low SIRI group (n=386)	High SIRI group (n=305)	P value
Hospital stay (day), median[IQR]	17.00[13.00,20.00]	18.76[16.00,26.88]	<0.001
ICU stay (day), median[IQR]	4.00[2.00,6.00]	5.00[3.76,15.21]	<0.001
Thoracic drainage (mL/48h), median[IQR]	740.00[530.00,1021.25]	775.00[520.00,1180.00]	0.097
Mechanical ventilation time (h), median[IQR]	42.00[24.00,75.00]	64.69[48.03,168.00]	<0.001
Need CRRT, (n, %)	31 (8.03)	68 (22.30)	<0.001
Need ECOM, (n, %)	1 (0.26)	13 (4.26)	<0.001
AKI (n, %)	39 (10.10)	92 (30.16)	<0.001
PND (n, %)	18 (4.66)	31 (10.16)	0.005
LCOS (n, %)	9 (2.33)	20 (6.56)	0.006
GB (n, %)	7 (1.81)	24 (7.87)	<0.001
Sepsis (n, %)	1 (0.26)	4 (1.31)	0.105
Secondary intubation (n, %)	20 (5.18)	38 (12.46)	<0.001
Tracheotomy (n, %)	15 (3.89)	32 (10.49)	<0.001
Secondary thoracotomy (n, %)	0	2 (0.66)	1.000
In-hospital mortality (%)	3 (0.78)	47 (15.41)	<0.001
MAEs (n, %)	52 (13.47)	123 (40.33)	<0.001
ARAEs (n, %)	22 (5.74)	91 (35.27)	<0.001

IQR, Interquartile range; CRRT, Continuous renal replacement therapy; ECOM, Extracorporeal membrane oxygenation; AKI, Acute renal injury; PND, Permanent neurological dysfunction; LCOS, Low cardiac output syndrome; GB, Gastrointestinal bleeding; MAEs, Major adverse events; ARAEs, Aorta-related adverse events.

## Discussion

For the extremely dangerous and poor-prognosis cardiovascular emergency known as AD, numerous treatment challenges still exist. While advancements in surgical techniques and extracorporeal circulation technology have contributed to improved treatment outcomes (11), multicenter studies have reported postoperative mortality rates as high as 10%-25% for AAAD (5), with medium- to long-term survival rates of only around 85% and 70% (5, 12–14). In clinical practice, the assessment of prognostic risk for AAAD patients largely depends on the clinical experience of the physician, as there is a lack of objective parameter indicators to provide valuable reference information. Therefore, finding simple and reliable preoperative biological indicators to assist clinical physicians in judging prognostic outcomes can better guide their clinical decision-making.

In this study, we discussed the association between SIRI and the prognosis of AAAD patients underwent emergency open surgical treatment. The research results indicate that SIRI is a key influencing factor for both short-term and long-term prognosis in AAAD patients. Elevated preoperative SIRI (>10.764) is closely associated with an increased rate of MAEs postoperatively as well as mid- to long-term ARAEs. Therefore, SIRI can serve as a simple and reliable biological marker for risk stratification and management decision-making in AAAD surgical patients.

AD is characterized by degenerative changes in the middle layer of the aortic wall, leading to weakening of the aortic wall. Inflammatory reactions and immune activation have been proven to play significant roles in this process (15–17). Many studies have reported the value of certain inflammatory biomarkers in the prognosis of AD. However, a single indicator often only reflects changes in a specific pathological pathway and cannot fully represent the complete pathophysiology of AD. Additionally, due to limitations in sample size, these studies cannot fully establish the correlation between inflammatory and immune factors and the prognosis outcomes of AD (18–20). Composite inflammatory indices, which are based on combinations of individual markers along the inflammatory pathophysiological axis, can more comprehensively reflect the results mediated by multiple pathological pathways (21, 22). Utilizing data from large sample sizes may provide a more reliable reference for predicting the prognosis outcomes of AD.

In recent years, SIRI has been identified as a novel composite biomarker that can comprehensively reflect systemic inflammation and immune status. Initially reported by Qi et al., its application in predicting the post-chemotherapy survival rate of pancreatic cancer patients was studied (23). As inflammatory responses and immune activation have been shown to play important roles in the development of cardiovascular diseases, SIRI has also gradually become a reliable indicator for predicting the prognosis of cardiovascular diseases (8, 24). Although we have found that elevated preoperative SIRI can be used to predict the postoperative prognosis of AAAD patients at different time points, the exact mechanisms remain unclear and require further research.

Compared to other inflammatory biomarkers, we believe that SIRI can more reliably predict the prognosis of AAAD due to the cellular functions of the three white blood cell subtypes that make up SIRI, including neutrophils, lymphocytes, and monocytes. Firstly, neutrophils, as an important cell type in the inflammatory response, not only secrete inflammatory mediators and regulate inflammation but also possess strong chemotactic and phagocytic abilities. They can adhere, engulf, and release various pro-inflammatory factors, thereby promoting vascular wall inflammation and damage (25). In contrast to the inflammatory activation caused by neutrophil activation, lymphocytes, as an important component of the immune system, primarily function to regulate and suppress the inflammatory response. Studies have reported that the presence of lymphocytes can alleviate inflammation, promote tissue repair, help maintain vascular wall integrity, and also regulate the activity of other immune cells and inhibit the release of inflammatory mediators, thereby reducing vascular wall inflammation and damage (26). Lymphocyte reduction has been shown to be associated with adverse outcomes in many cardiovascular diseases and is decreased in AAAD patients (27–29). As the main effector cells of the inflammatory response, monocytes are recruited from the bone marrow to the bloodstream and aortic tissue earliest during the initial stages of inflammation (30). Monocytes can activate the immune system through phagocytosis, chemotaxis, antigen presentation, and lymphocyte activation, determining the progression of systemic inflammation (31). These mechanisms may explain why SIRI can provide more prognostic information.

A higher SIRI may indicate more severe inflammatory response and a state of immune dysregulation in the body. The systemic inflammatory storm caused by AD releases a large number of inflammatory mediators and cytokines into the blood, negatively affecting multiple organs and potentially leading to early postoperative complications such as multiple organ dysfunction syndrome, increasing the incidence of MAEs during hospitalization. Inflammation also further promotes intraluminal thrombosis and fibrous tissue proliferation, leading to the progression of aortic dissection, while also increasing the fragility of the vascular wall and further increasing the risk of distal aortic dilation or even rupture, thereby affecting medium-term and long-term prognosis.

Currently, perioperative treatment strategies for patients undergoing AAAD surgery typically prioritize preoperative blood pressure control and maintaining hemodynamic stability, often overlooking the impact of systemic and local inflammatory states on prognosis. The application of SIRI may change this situation and further provide evidence-based support for perioperative management strategies for AAAD patients. Identifying high-risk patients early through SIRI can assist clinicians in stratifying preoperative risks and thereby assessing the risk levels of patients for postoperative MAEs. Additionally, for patients with elevated preoperative SIRI, more attention should be paid to the long-term prognosis after surgery, and more targeted follow-up measures should be implemented, such as increasing the frequency of follow-up examinations, incorporating inflammatory and immune markers into routine blood tests, and shortening the intervals for imaging examinations, in order to reduce the incidence of ARAEs.

Our study still has some limitations that need to be addressed: Firstly, this is a single-center retrospective study, which may introduce selection bias and information bias. Therefore, further rigorous multi-center prospective randomized controlled trials are needed to evaluate its external validity before it can be applied in clinical practice. Secondly, the specific mechanisms underlying the predictive value of SIRI levels for different timepoint outcomes are still unclear. Further research into its potential molecular and biological mechanisms may provide new insights. Additionally, although SIRI is a novel composite inflammatory and immune biomarker, when predicting outcome, it still needs to be combined with more perioperative clinical data, including hemodynamic indicators, use of vasoactive drugs, and imaging examination results, to better assist clinicians in decision-making and management. Nonetheless, our study results still provide important clinical evidence for the potential relationship between preoperative SIRI and outcomes at different timepoints in AAAD patients.

## Data availability statement

The original contributions presented in the study are included in the article/**Supplementary Material**. Further inquiries can be directed to the corresponding authors.

## Ethics statement

The studies involving humans were approved by Ethics Committee of Fujian Medical University Affiliated Union Hospital. The studies were conducted in accordance with the local legislation and institutional requirements. Written informed consent for participation was not required from the participants or the participants’ legal guardians/next of kin because Due to the retrospective nature of the study, informed consent was not required.

## Author contributions

LX: Writing – review & editing, Writing – original draft. QX: Writing – review & editing, Writing – original draft. WG: Writing – review & editing, Writing – original draft, Visualization, Validation, Data curation. QW: Writing – review & editing, Writing – original draft. XL: Writing – review & editing, Writing – original draft, Software, Data curation. ZQ: Writing – review & editing, Writing – original draft, Resources, Methodology. LC: Writing – review & editing, Writing – original draft, Resources, Methodology, Funding acquisition.
