# Supplementary material for: The prognostic value of preoperative systemic inflammatory response index in predicting outcomes of acute type A aortic dissection patients underwent surgical treatment
Source: Front Immunol. 2024 May 10;15:1388109. doi: 10.3389/fimmu.2024.1388109 (PMC11116625; doi:10.3389/fimmu.2024.1388109)
Supplement: Supplementary file 1 [file Table_1.docx]

| **Supplementary Table 1. Univariate analysis of variables associated with postoperative MAEs.** | | | |
| --- | --- | --- | --- |
| **Valuables** | **Non-MAEs group**  **(n=516)** | **MAEs group**  **(n=175)** | **P value** |
| **Demographical data** | | | |
| Age (years), median[IQR] | 53.00[44.00,62.00] | 55.00[47.00,65.00] | 0.102 |
| Gender (Male), n (%) | 323(62.60) | 115(65.71) | 0.459 |
| Body mass index (Kg/mˆ2), median[IQR] | 24.36[22.41,26.57] | 24.57[23.47,26.06] | 0.292 |
| LVEF (%), median[IQR] | 64.00[60.50,67.50] | 63.38[60.10,66.50] | 0.131 |
| **Risk factors and comorbidities** | | | |
| Smoking, n (%) | 240(46.51) | 69(39.43) | 0.103 |
| Alcohol, n (%) | 86(16.67) | 32(18.29) | 0.623 |
| Hypertension, n (%) | 380(73.64) | 131(74.86) | 0.752 |
| Diabetes, n (%) | 16(3.10) | 4(2.29) | 0.578 |
| Previous CAD, n (%) | 2(0.39) | 2(1.14) | 0.255 |
| Previous CVD, n (%) | 14(2.71) | 6(3.43) | 0.626 |
| Previous CKD, n (%) | 5(0.97) | 3(1.71) | 0.426 |
| Marfan Syndrome, n (%) | 14(2.71) | 1(0.57) | 0.093 |
| Pericardial effusion (Medium or above), n (%) | 35(6.78) | 17(9.71) | 0.204 |
| Aortic valve regurgitation (Medium or above), n (%) | 121(23.45) | 30(17.14) | 0.081 |
| **Preoperative laboratory results** | | | |
| WBC (×10ˆ9/L), median[IQR] | 11.84[9.43,14.35] | 13.57[10.66,16.95] | <0.001 |
| HB (g/L), median[IQR] | 132.00[121.00,143.00] | 129.00[117.00,142.00] | 0.068 |
| PLT (×10ˆ9/L), median[IQR] | 184.00[150.00,221.00] | 164.00[133.00,201.00] | <0.001 |
| ALT (IU/L), median[IQR] | 25.00[15.00,37.00] | 27.00[19.00,48.00] | 0.018 |
| AST (IU/L), median[IQR] | 25.00[19.00,40.00] | 28.00[19.00,65.00] | 0.124 |
| ALB (g/L), median[IQR] | 38.50[35.20,41.00] | 37.20[34.40,40.00] | 0.001 |
| Creatinine (μmol/L), median[IQR] | 75.00[63.00,102.10] | 84.00[58.00,142.00] | 0.013 |
| D-dimer (μg/mL), median[IQR] | 8.49[3.70,18.18] | 11.06[6.15,20.00] | <0.001 |
| Fibrinogen (g/L), median[IQR] | 2.79[2.04,4.01] | 2.35[1.67,3.00] | <0.001 |
| BNP (pg/mL), median[IQR] | 246.00[118.00,588.00] | 277.00[154.00,913.00] | 0.010 |
| NLR, median[IQR] | 12.45[7.90,17.39] | 15.99[11.83,22.74] | <0.001 |
| MLR, median[IQR] | 0.75[0.51,1.20] | 1.10[0.77,1.58] | <0.001 |
| PLR, median[IQR] | 208.99[153.29,305.33] | 230.86[157.83,306.15] | 0.472 |
| SII, median[IQR] | 2187.26[1343.25,3235.47] | 2623.74[1723.52,3799.27] | 0.003 |
| SIRI, median[IQR] | 8.49[4.44,13.62] | 13.46[9.36,17.71] | <0.001 |
| **Intraoperative conditions** | | | |
| Ascending aorta replacement, n (%) | 515(99.81) | 175(100.00) | 1.000 |
| **Root surgery** 0.256 | | | |
| Untreated | 199(38.57) | 58(33.14) |  |
| Reconstruction of sinus of valsava | 200(38.76) | 83(47.43) |  |
| Bentall | 104(20.16) | 27(15.43) |  |
| Wheat | 11(2.13) | 5(2.86) |  |
| David | 1(0.19) | 1(0.57) |  |
| CABG (n, %) | 16(3.10) | 8(4.57) | 0.359 |
| Mitral surgery (n, %) | 5(0.97) | 5(2.86) | 0.071 |
| TVP (n, %) | 7(1.36) | 4(2.29) | 0.396 |
| Operation time (min), median[IQR] | 245.00[225.52,279.00] | 257.00[232.48,296.00] | <0.001 |
| CPB time (min), median[IQR] | 149.00[137.00,168.00] | 155.00[139.19,188.00] | 0.002 |
| ACC time (min), median[IQR] | 60.00[48.00,75.00] | 60.91[49.00,83.00] | 0.121 |
| DHCA time (min), median[IQR] | 13.00[12.00,14.00] | 13.00[12.00,14.00] | 0.191 |
| Plasma transfusion volume (mL), median[IQR] | 250.00[200.00,350.00] | 250.00[200.00,400.00] | 0.850 |
| RBC transfusion volume (U), median[IQR] | 4.00[0.00,4.00] | 3.50[0.00,4.00] | 0.335 |
| Platelet transfusion volume (U), median[IQR] | 1.80[0.80,10.00] | 1.00[0.80,10.00] | 0.184 |
| IQR, Interquartile range; MAEs, Major adverse events; **LVEF**, Left ventricular ejection fraction; CAD, Coronary artery disease; CVD, Cerebrovascular disease; CKD, Chronic kidney disease; WBC, White blood cell; HB, Heamoglobin; PLT, Platelet; **ALT**, Alanine transaminase; **AST**, Aspartate transaminase; **ALB**, Albumin; BNP, B-type natriuretic peptide; **NLR**, Neutrophil-to-lymphocyte ratio; **MLR**, Monocyte-to-lymphocyte ratio; **PLR**, Platelet-to-lymphocyte ratio; **SII**, Systemic immune inflammation index; SIRI, Systemic inflammatory response index; **CABG**, Coronary artery bypass grafting; **TVP**, Tricuspid valvuloplasty; **CPB**, Cardiopulmonary bypass; **ACC**, Aortic cross clamp; **DHCA**, Deep hypothermic circulatory arrest; **RBC**, Red blood cell. | | | |
|  |  |  |  |
|  |  |  |  |
