# Supplementary material for: The prognostic value of preoperative systemic inflammatory response index in predicting outcomes of acute type A aortic dissection patients underwent surgical treatment
Source: Front Immunol. 2024 May 10;15:1388109. doi: 10.3389/fimmu.2024.1388109 (PMC11116625; doi:10.3389/fimmu.2024.1388109)
Supplement: Supplementary file 2 [file Table_2.docx]

| **Supplementary Table 2. Univariate analyses of variables associated with ARAEs** | | | |
| --- | --- | --- | --- |
| **Valuables** | **Non-ARAEs group**  **(n=528)** | **ARAEs group**  **(n=113)** | **P value** |
| **Demographical data** | | | |
| Age (years), median[IQR] | 53.00[44.00,62.00] | 55.00[49.00,64.00] | 0.238 |
| Gender (Male), n (%) | 327(61.93) | 80(70.80) | 0.076 |
| Body mass index (Kg/mˆ2), median[IQR] | 24.36[22.49,26.57] | 24.84[23.34,25.95] | 0.420 |
| LVEF (%), median[IQR] | 64.03[60.40,67.40] | 63.30[60.10,66.30] | 0.369 |
| **Risk factors and comorbidities** | | | |
| Smoking, n (%) | 239(45.27) | 46(40.71) | 0.376 |
| Alcohol, n (%) | 90(17.05) | 18(15.93) | 0.774 |
| Hypertension, n (%) | 390(73.86) | 83(73.45) | 0.928 |
| Diabetes, n (%) | 13(2.46) | 6(5.31) | 0.105 |
| Previous CAD, n (%) | 2(0.38) | 1(0.89) | 0.474 |
| Previous CVD, n (%) | 15(2.84) | 3(2.66) | 0.913 |
| Previous CKD, n (%) | 7(1.33) | 1(0.89) | 0.702 |
| Marfan Syndrome, n (%) | 12(2.27) | 3(2.66) | 0.807 |
| Pericardial effusion (Medium or above), n (%) | 40(7.58) | 6(5.31) | 0.397 |
| Aortic valve regurgitation (Medium or above), n (%) | 123(23.30) | 20(17.70) | 0.195 |
| **Preoperative laboratory results** | | | |
| WBC (×10ˆ9/L), median[IQR] | 11.73[9.43,14.29] | 13.86[11.11,17.57] | <0.001 |
| HB (g/L), median[IQR] | 131.00[119.00,142.00] | 132.00[122.66,144.00] | 0.461 |
| PLT (×10ˆ9/L), median[IQR] | 181.00[148.00,216.00] | 177.00[135.00,218.00] | 0.377 |
| ALT (IU/L), median[IQR] | 25.00[16.00,38.00] | 27.00[17.00,50.00] | 0.139 |
| AST (IU/L), median[IQR] | 25.00[19.00,41.00] | 26.00[18.00,69.00] | 0.630 |
| ALB (g/L), median[IQR] | 38.20[35.00,40.90] | 37.90[34.90,40.20] | 0.358 |
| Creatinine (μmol/L), median[IQR] | 77.00[63.00,105.00] | 81.00[60.00,132.00] | 0.213 |
| D-dimer (μg/mL), median[IQR] | 8.77[3.72,18.51] | 10.85[5.49,20.00] | 0.012 |
| Fibrinogen (g/L), median[IQR] | 2.72[2.03,3.95] | 2.40[1.77,3.19] | 0.007 |
| BNP (pg/mL), median[IQR] | 256.00[119.00,630.00] | 281.00[140.00,572.00] | 0.318 |
| NLR, median[IQR] | 12.10[7.76,16.61] | 17.96[12.99,27.24] | <0.001 |
| MLR, median[IQR] | 0.72[0.51,1.08] | 1.36[0.96,1.79] | <0.001 |
| PLR, median[IQR] | 205.05[146.84,283.33] | 259.74[177.08,390.43] | <0.001 |
| SII, median[IQR] | 2078.38[1300.66,3087.09] | 3158.49[2061.71,4933.85] | <0.001 |
| SIRI, median[IQR] | 7.81[4.36,12.22] | 15.01[9.90,24.18] | <0.001 |
| **Intraoperative conditions** | | | |
| Ascending aorta replacement, n (%) | 527(99.81) | 113(100.00) | 1.000 |
| **Root surgery** 0.687 | | | |
| Untreated | 198(37.50) | 40(35.40) |  |
| Reconstruction of sinus of valsava | 212(40.15) | 51(45.13) |  |
| Bentall | 104(19.70) | 18(15.93) |  |
| Wheat | 12(2.27) | 3(2.66) |  |
| David | 1(0.19) | 1(0.89) |  |
| CABG (n, %) | 15(2.84) | 4(3.54) | 0.691 |
| Mitral surgery (n, %) | 6(1.14) | 1(0.89) | 0.815 |
| TVP (n, %) | 8(1.52) | 2(1.77) | 0.843 |
| Operation time (min), median[IQR] | 245.23[225.87,279.00] | 254.00[228.67,286.00] | 0.139 |
| CPB time (min), median[IQR] | 149.68[137.16,168.00] | 153.00[137.00,176.00] | 0.368 |
| ACC time (min), median[IQR] | 60.00[48.00,75.00] | 60.00[48.00,78.00] | 0.479 |
| DHCA time (min), median[IQR] | 13.00[12.00,14.00] | 13.00[12.00,13.00] | 0.691 |
| Plasma transfusion volume (mL), median[IQR] | 250.00[200.00,350.00] | 200.00[0,400.00] | 0.541 |
| RBC transfusion volume (U), median[IQR] | 4.00[0,4.00] | 4.00[0,4.00] | 0.385 |
| Platelet transfusion volume (U), median[IQR] | 1.50[0.80,10.00] | 1.80[0.80,10.00] | 0.706 |
| IQR, Interquartile range; **ARAEs**, aorta-related adverse events; **LVEF**, Left ventricular ejection fraction; CAD, Coronary artery disease; CVD, Cerebrovascular disease; CKD, Chronic kidney disease; WBC, White blood cell; HB, Heamoglobin; PLT, Platelet; **ALT**, Alanine transaminase; **AST**, Aspartate transaminase; **ALB**, Albumin; BNP, B-type natriuretic peptide; **NLR**, Neutrophil-to-lymphocyte ratio; **MLR**, Monocyte-to-lymphocyte ratio; **PLR**, Platelet-to-lymphocyte ratio; **SII**, Systemic immune inflammation index; SIRI, Systemic inflammatory response index; **CABG**, Coronary artery bypass grafting; **TVP**, Tricuspid valvuloplasty; **CPB**, Cardiopulmonary bypass; **ACC**, Aortic cross clamp; **DHCA**, Deep hypothermic circulatory arrest; **RBC**, Red blood cell. | | | |
|  |  |  |  |
|  |  |  |  |
