# Supplementary material for: The prognostic value of preoperative systemic inflammatory response index in predicting outcomes of acute type A aortic dissection patients underwent surgical treatment
Source: Front Immunol. 2024 May 10;15:1388109. doi: 10.3389/fimmu.2024.1388109 (PMC11116625; doi:10.3389/fimmu.2024.1388109)
Supplement: Supplementary file 3 [file Table_3.docx]

| **Supplementary Table 3**. **Receiver operating characteristic curve analysis for postoperative MAEs** | | | | |
| --- | --- | --- | --- | --- |
| Valuables | AUC | Cut-off value | **Sensitivity** | **Specificity** |
| NLR | 0.638 | 13.413 | 0.663 | 0.566 |
| MLR | 0.679 | 0.773 | 0.749 | 0.519 |
| PLR | 0.518 | 237.255 | 0.497 | 0.581 |
| SII | 0.576 | 2783.016 | 0.480 | 0.665 |
| SIRI | 0.698 | 10.764 | 0.703 | 0.647 |
| MAEs, Major adverse events; **AUC**, the area under the receiver operating characteristic curve; **NLR**, Neutrophil-to-lymphocyte ratio; **MLR**, Monocyte-to-lymphocyte ratio; **PLR**, Platelet-to-lymphocyte ratio; **SII**, Systemic immune inflammation index; **SIRI**, Systemic inflammatory response index. | | | | |
