# Supplementary material for: The prognostic value of preoperative systemic inflammatory response index in predicting outcomes of acute type A aortic dissection patients underwent surgical treatment
Source: Front Immunol. 2024 May 10;15:1388109. doi: 10.3389/fimmu.2024.1388109 (PMC11116625; doi:10.3389/fimmu.2024.1388109)
Supplement: Supplementary file 4 [file Table_4.docx]

| **Supplementary Table 4**. **Receiver operating characteristic curve analysis for ARAEs** | | | | |
| --- | --- | --- | --- | --- |
| Valuables | AUC | Cut-off value | **Sensitivity** | **Specificity** |
| NLR | 0.733 | 14.198 | 0.712 | 0.633 |
| MLR | 0.789 | 0.892 | 0.822 | 0.650 |
| PLR | 0.621 | 237.255 | 0.607 | 0.614 |
| SII | 0.690 | 3087.391 | 0.540 | 0.750 |
| SIRI | 0.822 | 10.764 | 0.847 | 0.684 |
| **ARAEs**, aorta-related adverse events; **AUC**, the area under the receiver operating characteristic curve; **NLR**, Neutrophil-to-lymphocyte ratio; **MLR**, Monocyte-to-lymphocyte ratio; **PLR**, Platelet-to-lymphocyte ratio; **SII**, Systemic immune inflammation index; **SIRI**, Systemic inflammatory response index. | | | | |
